# Diagnostic and Therapeutic Aspects of Monoclonal Gammopathies of Renal Significance (MGRS): An Update

**DOI:** 10.3390/diagnostics14242892

**Published:** 2024-12-23

**Authors:** Giuseppe Stefano Netti, Dario Troise, Michele Rossini, Valeria Catalano, Federica De Luca, Javeria Khalid, Valentina Camporeale, Fabiana Ritrovato, Barbara Infante, Francesca Sanguedolce, Giovanni Stallone, Elena Ranieri

**Affiliations:** 1 Unit of Clinical Pathology, Department of Medical and Surgical Sciences, University of Foggia, University Hospital “Policlinico Riuniti”, Viale Luigi Pinto, 71122 Foggia, Italy; valeria.catalano@unifg.it (V.C.); federica.deluca@unifg.it (F.D.L.); javeria.khalid@unifg.it (J.K.); valentina.camporeale@unifg.it (V.C.); fabiana.ritrovato@unifg.it (F.R.); elena.ranieri@unifg.it (E.R.); 2 Center for Research and Innovation in Medicine (CREATE), Department of Medical and Surgical Sciences, University of Foggia, University Hospital “Policlinico Riuniti”, Viale Luigi Pinto, 71122 Foggia, Italy; dario.troise@unifg.it (D.T.); giovanni.stallone@unifg.it (G.S.); 3 Unit of Nephrology, Dialysis and Transplantation, Advanced Research Center on Kidney Aging (A.R.K.A.), Department of Medical and Surgical Sciences, University of Foggia, University Hospital “Policlinico Riuniti”, Viale Luigi Pinto, 71122 Foggia, Italy; clafne@gmail.com; 4 Unit of Nephrology, Dialysis and Transplantation, Department of Precision and Regenerative Medicine and Ionian Area (DiMePRe-J), University of Bari “Aldo Moro”, Piazza Giulio Cesare 11, 70124 Bari, Italy; michelerossini@uniba.it; 5 Unit of Pathology, Department of Clinical and Experimental Medicine, University of Foggia, University Hospital “Policlinico Riuniti”, Viale Luigi Pinto, 71122 Foggia, Italy; francesca.sanguedolce@unifg.it

**Keywords:** monoclonal gammopathies, lymphoproliferative disorders, paraprotein-related renal disease, chronic kidney disease, light-chains, renal biopsy

## Abstract

Monoclonal gammopathy of renal significance (MGRS) refers to a group of renal disorders caused by a monoclonal immunoglobulin (MIg), secreted by a non-malignant B-cell clone. Unlike overt multiple myeloma or B-cell proliferation, MGRS does not meet those diagnostic criteria. However, it is associated with significant morbidity, due to severe renal, and sometimes systemic, lesions induced by the MIg. Early recognition is crucial, as chemotherapy to suppress MIg secretion often improves outcomes. The spectrum of renal diseases in MGRS is broad, including both well-known conditions like AL amyloidosis and newly described lesions. Kidney biopsy is essential to determine the specific lesion associated with MGRS and assess its severity. Diagnosis involves integrating morphologic alterations using techniques such as light microscopy, immunofluorescence (IF), electron microscopy, and, in some cases, IF staining for Ig isotypes, immunoelectron microscopy, and proteomic analysis. Additionally, a complete hematologic evaluation, including serum and urine protein electrophoresis, immunofixation, and a serum-free light-chain assay, is necessary.

## 1. Introduction

Paraproteins are monoclonal immunoglobulins (Ig), or their components, that are produced by clones of B-cells. Monoclonal gammopathy (MG) can appear in any of the five immunoglobulin isotypes (IgA, IgG, IgM, IgD, and IgE). IgG, which is the most abundant among all immunoglobulins, also represents the most common form of MG, while IgE is the rarest [1]. In approximately 0.7–0.8% of the population over the age of 45, monoclonal gammopathies involving only the light-chains of immunoglobulins are found [2,3,4]. Plasma cells may release all five Ig isotypes, whereas less-differentiated B-cells typically have a limited capacity to synthesize all isotypes. Clones with lymphoplasmacytic differentiation (Waldenström) almost always produce IgM, while CD51^+^ CD231^+^ lymphocytes (chronic lymphocytic leukemia or CLL) generally release IgG and, rarely, IgA [5].

Detecting monoclonal gammopathy serves as a biomarker indicating the clonal proliferation of cells that can produce monoclonal immunoglobulins. In plasma cell dyscrasias, this finding can be linked to a broad range of hematological conditions, ranging from clinically silent forms to highly aggressive ones (Table 1) [6].

The progression of these diseases is typically marked by an increase in the measurable monoclonal component in the serum, either as a monoclonal spike on electrophoresis or as the concentration of free light-chains in the serum (serum-free light-chain, sFLC) [7].

The two most common clinical scenarios occur during the progression of multiple myeloma and Waldenström macroglobulinemia. In the first case, as the concentration of free light-chains increases, so too does the risk of intratubular cylinder formation, capable of causing light-chain cast nephropathy (LCCN), which is, by definition, related to multiple myeloma [8]. The second scenario is characterized by hyperviscosity syndrome, due to high levels of monoclonal proteins, most commonly seen in Waldenström macroglobulinemia, although it can also occur in multiple myeloma [9]. These MG-related events imply the presence of a tumor mass that increases the risk of organ damage.

However, the paraprotein can be nephrotoxic and it directly contributes to the development of kidney diseases, regardless of its concentration or the size of the tumor mass. In these conditions, the progression of the neoplastic clone is not necessary. In fact, in these cases, the features of the monoclonal cell line are much more similar to MGUS, a “smoldering” form of Waldenström macroglobulinemia (WM), or monoclonal B-cell lymphocytosis, than to their respective malignant variants. The term ’monoclonal gammopathy of renal significance’ (MGRS) was introduced by the International Kidney and Monoclonal Gammopathy Research Group to encompass all proliferative disorders of B lymphocytes and plasma cells that do not meet the diagnostic criteria for MM, WM, CLL, or malignant lymphoma, but are related to the development of nephropathies that are related to the presence of a paraprotein [10].

In humans, the nephrotoxicity of paraproteins is evident in light-chain amyloidosis (AL), where 40% of patients have more than 10% of plasma cells in the bone marrow, but less than 20% meet the criteria for the diagnosis of multiple myeloma (MM) [6]. Similarly, only 20–60% of patients with Monoclonal Ig Deposition Disease (MIDD) meet the criteria for MM diagnosis; in most patients, it is caused by the deposition of kappa light-chains and carries a high risk of developing End-Stage Renal Disease (ESRD) [11].

Previously, prior to the definition of MGRS, these conditions were not encompassed within the clonal disorders, and were, therefore, not treated with therapies available for myeloma or lymphomas. Consequently, a poor clinical response and the progression of chronic kidney disease were observed [12,13,14]. The correct classification of these diseases has improved the understanding of pathogenetic mechanisms, enabling the development, targeted treatment strategies, and improved prognosis [11,15].

## 2. Pathogenesis of Renal Lesions Associated with MGRS

Many kidney diseases are linked to the deposition or precipitation of monoclonal immunoglobulins (Ig). These lesions are common in patients with paraproteinemia, and are linked to high rates of morbidity and mortality. Further, the clinical manifestations, histological lesions, involvement of other organs, and prognosis of renal lesions in the context of MGRS can vary widely. Many of these manifestations are likely determined by the type and rate of synthesis of the pathogenic monoclonal immunoglobulin, along with the local microenvironment [16].

In patients with MGRS, the deposition of monoclonal light-chains of immunoglobulins is predominantly observed, while deposition of the entire immunoglobulin is less common, and deposition of heavy-chains is extremely rare. The deposition of monoclonal components can affect various renal structures, including glomeruli, tubule-interstitium, and blood vessels [11,15,17,18]. Glomerular capillaries and the mesangium are the preferential sites for the deposition of monoclonal Ig [18]. Regardless of the underlying pathology, renal damage in the context of MGRS can be attributed to different pathogenetic mechanisms (Table 2).

**Figure 1:**
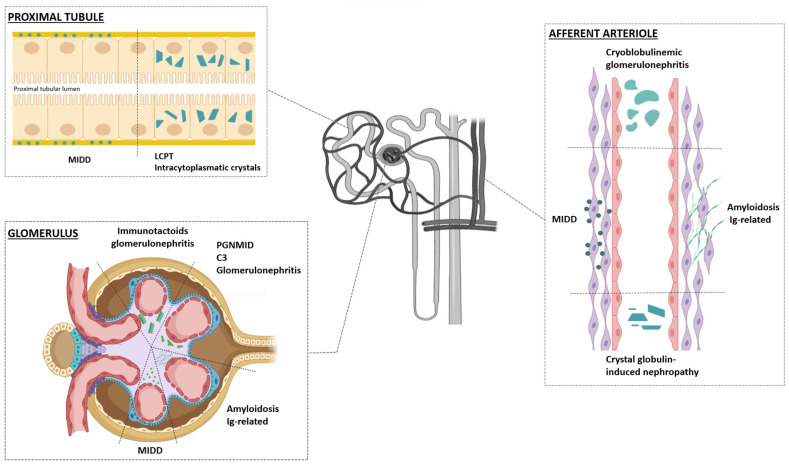
Localization of renal lesions in monoclonal gammopathy of renal significance (MGRS). Renal lesions associated with MGRS can include one or more renal compartments. In immunotactoid glomerulonephritis, C3 Glomerulopathy, and Proliferative Glomerulonephritis with Monoclonal Immunoglobulin Deposits (PGNMID), MGRS-associated lesions affect only the glomeruli, whereas in light-chain proximal tubulopathy (LCPT), the lesions involve only the proximal tubules. MGRS-associated lesions in cryoglobulinemic glomerulonephritis mainly involve the glomeruli, but can occasionally affect blood vessels (intravascular cryoglobulins or endovascular Ig-related vasculitis). Amyloidosis and monoclonal immunoglobulin deposition disease (MIDD) usually affect all renal compartments (glomeruli, vessels, and tubule-interstitium).

The pathogenetic role of free light-chains produced by the anomalous clone in the development of nephropathies varies according to the site of deposition (Figure 1). In detail, the free light-chains may interact directly with the kidney structures. Complex interactions between the biochemical characteristics of light-chains, especially in their variable region (glycosylation, hydrophobic residue insertion, etc.), and the glomerulo-tubular microenvironment (receptor systems, uromodulin or Tamm–Horsfall protein, growth factors, etc.) determine the site of renal damage, whether it is glomerular or tubular, and, consequently, the predominant type of histologically documented lesions [18,19].

Moreover, the kidney plays a key role in the physiological metabolism of free monoclonal light-chains (FLC). The serum concentration of FLC is determined by the equilibrium between their production by plasma cells and their elimination by the kidneys. Under normal conditions, the plasma concentration of κ FLC (molecular weight 22–25 kDa) ranges from 3.3–19.4 mg/L, while for λ FLC it is 5.7–26.3 mg/L. Determining the serum concentration of FLC is important to establish monoclonality based on the κ/λ ratio. This ratio ranges from 0.26–1.65 in patients with normal renal function. A ratio above 1.65 indicates monoclonality for κ chains, whereas a ratio below 0.26 indicates monoclonality for λ chains [11,18,19].

Finally, FLCs are rapidly cleared from the serum (half-life of 2–4 h) and metabolized in the renal proximal tubule. Only a small amount is excreted in the urine. In a day, the kidney can metabolize 10–30 g of FLC compared to a daily production of 0.5–1.0 g under physiological conditions [11].

Approximately 90% of circulating FLCs, due to their low molecular weight and positive charge, are freely filtered by the Glomerulus and subsequently reabsorbed in the proximal tubule, where FLCs undergo degradation processes. Tubular reabsorption and catabolism processes are mediated by a transport receptor system located at the brush-border of tubular cells. The cubilin/megalin system binds FLCs, which are then internalized into the tubular cell through clathrin-mediated endocytosis, and subsequently degraded by lysosomal hydrolytic enzymes [19,20].

**Figure 2:**
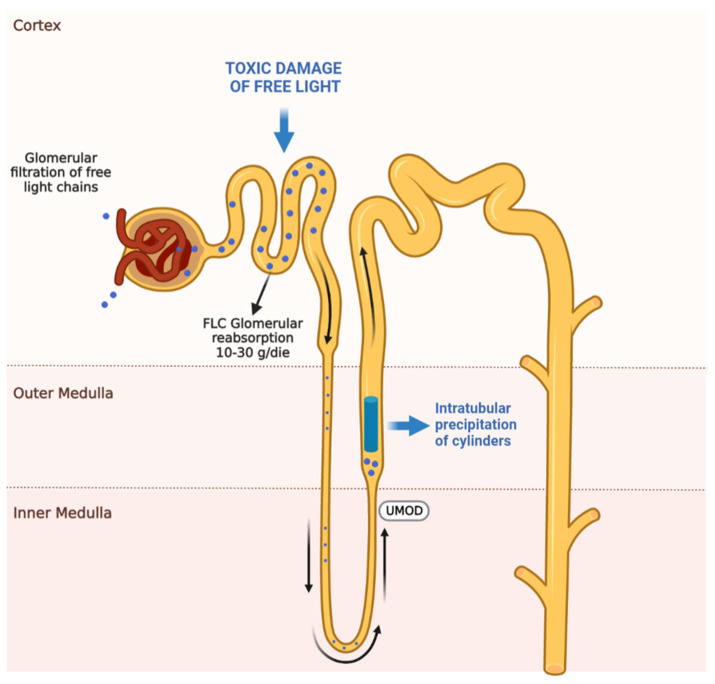
Pathogenesis of renal damage induced by light-chains in monoclonal gammopathy of renal significance (MGRS).

When the receptor-mediated reabsorption process reaches saturation, FLCs reach the distal tubule and are excreted in the urine at a high concentration. However, the production of pathological quantities of FLCs can exceed the reabsorption capacity of the proximal tubule, leading to an excess of FLCs in the distal tubules. These excess FLCs can bind to Tamm–Horsfall protein, a glycoprotein secreted by the thick ascending limb of the loop of Henle, inducing the formation of casts (Figure 2). Additionally, when the tubular catabolic process becomes overwhelmed, an excessive release of lysosomal enzymes may occur; furthermore, the tubular catalytic process, when saturated, can itself trigger an excessive release of lysosomal enzymes that lead to acute alteration of tubular cells with vacuolization, fragmentation, and desquamation [19,20].

Under pathological conditions, both the renal metabolism of FLCs and the different nephron sites where they exert their pathogenic effect depict the following two possible frameworks: glomerulopathic light-chains and tubulopathic light-chains [20].

The former framework, glomerulopathic, targets the mesangium, inducing various types of glomerular lesions that are determined by the ultrastructural characteristics of the deposits. The latter framework, termed tubulopathic, exerts its pathogenic action at the tubular level, both proximal and distal, leading to two distinct “tubulopathies”, namely Fanconi Syndrome and Myeloma Cast Nephropathy.

MGRS with glomerular lesions can be divided into two categories, based on the ultrastructural characteristics of their deposits. Glomerulopathies with organized deposits include AL amyloidosis, cryoglobulinemic glomerulonephritis types 1 and 2, and immunotactoid glomerulonephritis (also known as GN with microtubular-organized deposits of Ig). Glomerulopathies with unorganized deposits include Monoclonal Ig Deposition Disease (MIDD), Monoclonal Ig Proliferative Glomerulonephritis (PGNMID), and MG-associated C3 Glomerulopathy (MG-C3GN) [11,20].

**Figure 3:**
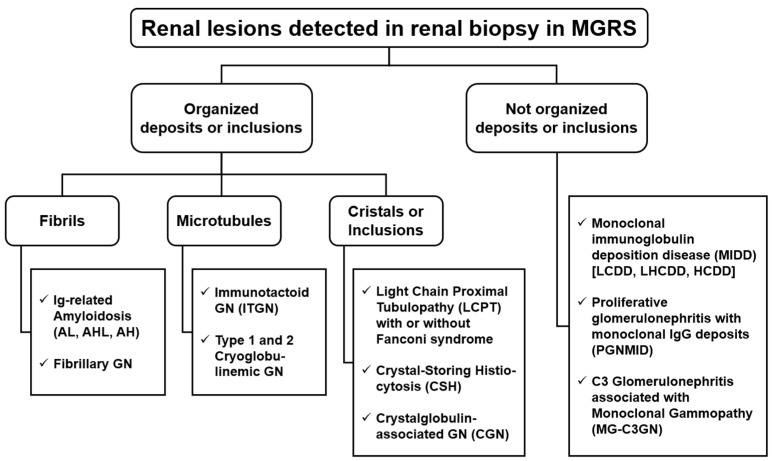
Histological lesions on renal biopsy in monoclonal gammopathy of renal significance (MGRS). Renal lesions associated with MGRS are firstly categorized according to the presence or absence of monoclonal immunoglobulin deposits on renal biopsy. These lesions are further divided based on the ultrastructural characteristics of the deposits, which can be either organized or unorganized. Organized deposits are further classified as fibrillar, microtubular, or inclusions or crystals. HCDD, heavy-chain deposition disease; LCDD, light-chain deposition disease; LHCDD, light- and heavy-chain deposition disease.

MGRS, plasmacytomas, and lymphomas can also lead to tubulointerstitial diseases, including light-chain proximal tubulopathy (LCPT), with or without Fanconi Syndrome, and Light-Chain Cast Nephropathy (LCCN). The latter is not strictly a lesion related to MGRS, as it is almost always secondary to multiple myeloma (MM). The renal lesions associated with MGRS are schematically described in Figure 1 and summarized in Figure 3 and Table 3 [11,20].

**Figure 4:**
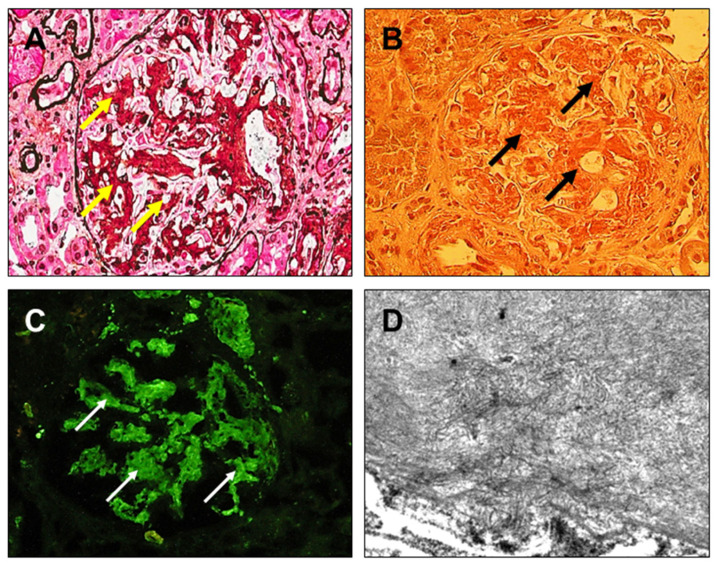
AL amyloidosis. (**A**) Glomerulus characterized by mesangial expansion due to accumulation of hypocellular material, weakly silver-positive (yellow arrows) [Silver-Methenamine, ×200]. (**B**) Accumulation of Congo-red-positive acellular material (black arrows) [hematoxylin eosin, ×200]. (**C**) Positive immunofluorescence for lambda-type light-chains with a homogeneous, predominantly mesangial pattern (white thin arrows) (×200). (**D**) On high magnification at the ultrastructural level, presence of fibrillar material, mainly in the mesangial area [electron microscopy, ×46,000].

**Figure 5:**
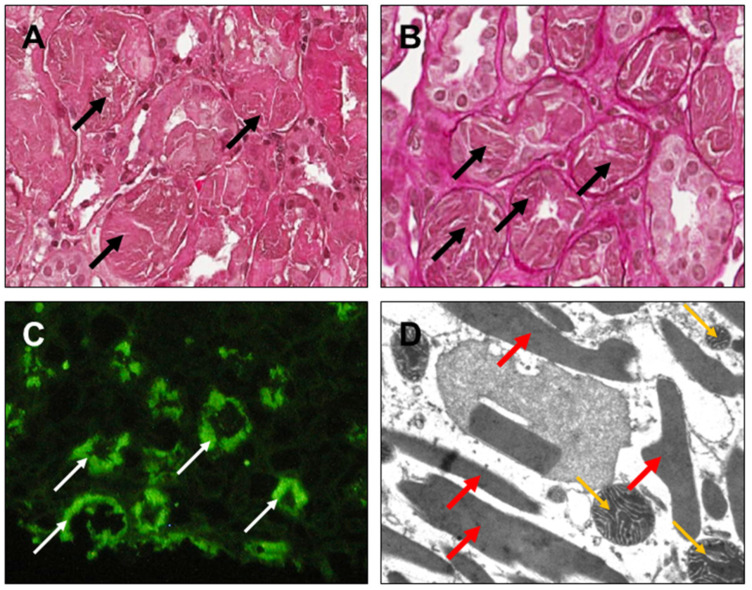
Light-chain proximal tubulopathy (LCPT). (**A**) Numerous proximal tubules showing cytoplasm filled with weakly PAS-positive protein material (black arrows) [hematoxylin eosin, ×200]. (**B**) Diffuse intracytoplasmic protein inclusions (black arrows) in proximal tubules [PAS, ×200]. (**C**) Positive diffuse immunofluorescence for tubular intracytoplasmatic kappa-type light-chains (white thin arrows) [×100]. (**D**) On high magnification at the ultrastructural level, amorphous granular accumulation, sometimes in crystalline form, of light-chains within the proximal tubular cells (red arrows); increased volume of lysosomes with a speckled appearance (yellow thin arrows) [electron microscopy, ×46,000].

**Figure 6:**
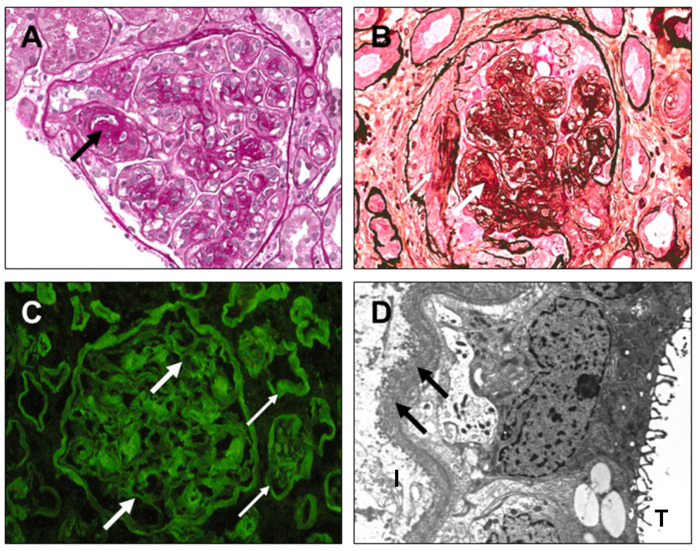
Monoclonal immunoglobulin deposition disease (MIDD). (**A**) Glomerulus with lobulated appearance, endocapillary hypercellularity, and presence of PAS-positive mesangial nodules (black arrow) [PAS, ×200]. (**B**) Glomerulus with lobulated appearance, endocapillary hypercellularity, double contours of the basement membranes, fibrous crescent (white thin arrow), and silver-positive mesangial nodules (white thick arrow) [Silver-Methenamin, ×200]. (**C**) At immunofluorescence, deposits of kappa light-chains at the glomerular level (homogeneous and linear mesangial along the capillary basement membranes) (white thick arrows) and along the tubular basement membranes (linear) (white thin arrows) [×200]. (**D**) On high magnification at the ultrastructural level, electron-dense deposits with “salt and pepper” appearance on the external side of the tubular basement membranes (black arrows). IS, interstitial space; TL, tubular lumen [electron microscopy, ×46,000].

**Figure 7:**
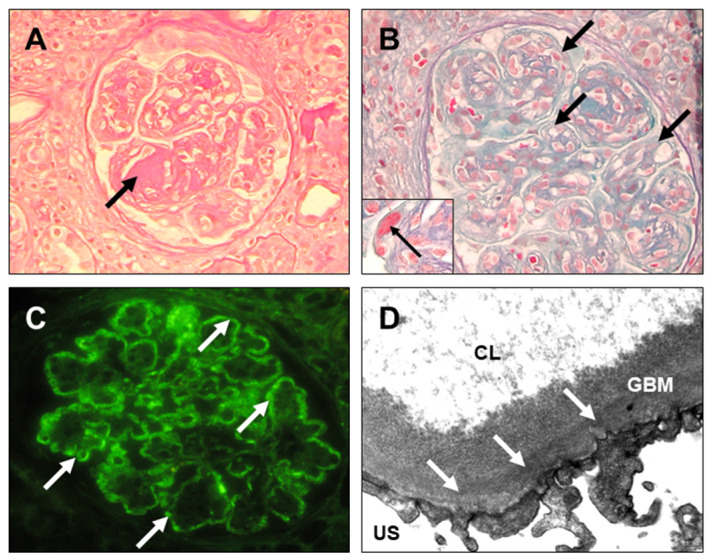
Proliferative Glomerulonephritis with Monoclonal IgG Deposits (PGNMID). (**A**) A Glomerulus with lobulated appearance and presence of a mesangial nodule (black arrow), which is strongly PAS-positive [PAS, ×200]. (**B**) Glomerulus with lobulated appearance and numerous double contours of the basement membranes (black thick arrows) associated with cellular interposition (membrane-proliferative pattern). There is also an increase in the matrix and mesangial cellularity [Trichrome, ×400]. Presence of double contours, cellular interposition, and jaline deposits (black thin arrow) in the thickness of the membranes [Trichrome, ×1000]. (**C**) At immunofluorescence, ribbon-like IgG deposits along the glomerular basement membranes [×400]. (**D**) On high magnification at the ultrastructural level, subendothelial electron-dense deposits (white arrows). CL, capillary lumen; GBM, glomerular basement membrane; US, urinary space [electron microscopy, ×46,000].

Furthermore, in Figure 4, Figure 5, Figure 6 and Figure 7, clinical cases of AL amyloidosis (Figure 4), light-chain proximal tubulopathy (LCPT) (Figure 5), monoclonal immunoglobulin deposition disease (MIDD) (Figure 6), and Proliferative Glomerulonephritis with Monoclonal IgG Deposits (PGNMID) (Figure 7) are shown and described (images are kindly provided by Dr. Michele Rossini, Nephropathology Laboratory, University Hospital “Policlinico”, Bari, Italy).

## 3. Clinical Features of Renal Disease Associated with MGRS

The spectrum of renal lesions in MGRS is broad (Table 3). MGRS is mostly observed in patients over 50 years of age, and the gender distribution seems to vary depending on the specific disorder. Glomerulopathies associated with MGRS are associated with a progressive decline in renal function, and a considerable number of patients proceed to end-stage renal disease (ESRD). The extent of proteinuria can vary from mild to frank nephrotic syndrome. Hematuria is usually microscopic, and hypertension is often present [11,21,22,23]. Cryoglobulinemic GN type 1 can also be associated with an acute deterioration of renal function associated with a nephritic picture [11,24]. Amyloidosis is less frequently associated with hematuria and hypertension, but is strongly correlated with nephrotic-range proteinuria [13,25,26]. Extrarenal manifestations are common in AL amyloidosis, cryoglobulinemic GN type 1, and MIDD, and can affect multiple organs to varying degrees, including the heart, liver, lungs, skin, joints, and peripheral nerves [11,20,24,26].

Tubular disorders may manifest with different levels of progressive chronic kidney disease, tubular proteinuria, and proximal tubular dysfunction, such as glycosuria, phosphaturia, and type 2 renal tubular acidosis [11,20]. Extrarenal manifestations, such as osteomalacia, may be linked to Fanconi Syndrome due to urinary phosphate loss [27]. Crystal accumulation histiocytosis can affect the hematopoietic bone marrow, liver, spleen, lymph nodes, lungs, skin, and cornea [11,20].

Renal disorders associated with MGRS have a high recurrence rate in the transplanted kidney. Reported recurrence rates in the literature for MIDD, for example, exceed 80% of cases [28,29]. Although fibrillary GN has been observed in <20% of patients with MG, the presence of MG can significantly increase the risk of post-transplant recurrence [30]. However, the time to recurrence varies between different diseases. The recurrence of lesions such as MIDD and PGNMID can be very rapid, occurring within weeks or months, while for other lesions, such as AL amyloidosis, several years may pass [22,31,32,33,34]. Clearance of monoclonal protein achieved through chemotherapy or autologous hematopoietic stem cell transplantation (ASCT) can significantly prevent or delay disease recurrence in the transplanted kidney [31,32,35,36].

## 4. Diagnosis of Renal Disease Associated with MGRS

**Figure 8:**
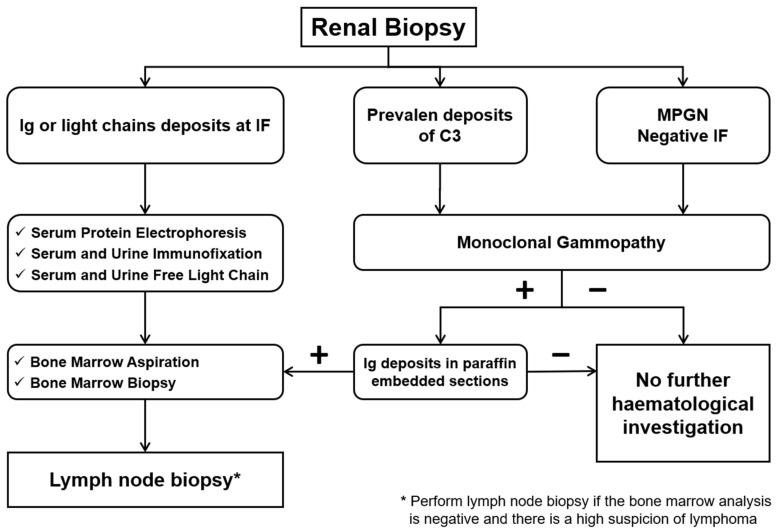
Diagnostic algorithm for hematologic disease if renal biopsy shows renal lesions related to monoclonal gammopathy of renal significance (MGRS).

Diagnosing renal disease associated with MGRS may be difficult due to the spectrum of renal manifestations, and detecting the pathogenicity of Ig is challenging in itself. This condition is further complicated by the absence of renal lesions from the monoclonal component in most patients with MG; instead, typical lesions of the elderly population (nephroangiosclerotic, diabetic, ischemic lesions) are observed. On the other hand, the search for a monoclonal component may be crucial in patients with unexplained proteinuria or chronic kidney disease, especially if they are over 50 years old. In patients in whom a monoclonal component is occasionally detected, it is essential to investigate renal involvement, which can manifest as a progressive loss of renal function or proteinuria. Similarly, renal biopsies may reveal deposits of monoclonal proteins [11,20,37]. The diagnostic algorithm, starting from renal biopsy, to correctly characterize the lesion and guide a detailed hematological investigation is shown in Figure 3 and Figure 8.

In almost all circumstances, when a monoclonal Ig is detected, if urinary abnormalities (proteinuria and/or hematuria) and/or a reduced GFR are detected, a renal biopsy should always be performed. As for all patients who undergo renal biopsy, the risks of bleeding related to the procedure in this cohort of patients must be carefully evaluated (i.e., poorly controlled higher blood pressure, shrunk kidney due to end-stage nephropathies). Renal biopsy has been demonstrated to be very safe in this patient group, with a complication rate comparable to that of patients undergoing renal biopsy for other kidney diseases [38]. Renal biopsy should not be restricted to patients with nephrotic-range proteinuria, as individuals with AL amyloidosis or light-chain deposition disease (LCDD) may exhibit predominantly tubular and vascular immunoglobulin deposition, with a urinary protein excretion below 0.5 g/day [39,40].

Immunofluorescence and ultrastructural studies using electron microscopy are essential to characterize the specific type of immunoglobulin deposits, their distribution patterns, and ultrastructural features (fibrillar, microtubular, or non-organized) [20].

Recently, it was discovered that immunoglobulins may not be detectable by immunofluorescence on frozen tissue. Instead, they can be “unmasked” using immunofluorescence on formalin-fixed, paraffin-embedded sections after protease digestion. Therefore, all patients with monoclonal gammopathy, in whom the biopsy has shown a pattern compatible with C3GN or immunofluorescence-negative membranoproliferative glomerulonephritis, should undergo immunofluorescence on paraffin-embedded tissue [20,41,42].

A crucial aspect of renal biopsy is correlating the specific immunoglobulin (Ig) identified in the biopsy with that found during hematological investigation. This ensures a direct link between the monoclonal gammopathy (MG) and the type of nephropathy (Figure 8).

According to the International Myeloma Working Group updated criteria, serum protein electrophoresis (SPEP) and serum protein immunofixation electrophoresis (SIFE) are commonly used as screening tests in the diagnosis of monoclonal gammopathies, and the findings of SIFE are currently the gold standard for identification of monoclonal immunoglobulins, including both intact immunoglobulins as well as free light-chains. Additional investigations recommended by the International Myeloma Workshop Consensus Panel 3 are serum-free light-chain quantification (SFLC), urine protein electrophoresis (UPEP), and urine immunofixation electrophoresis (UIFE).

The search for monoclonal Ig should begin with serum and urine protein electrophoresis, coupled with serum and urine immunofixation, to identify the Ig isotype and increase the sensitivity of detecting the monoclonal Ig. It is crucial to perform these analyses on both serum and urine because this approach significantly improves diagnostic sensitivity, especially in patients with AL amyloidosis or a small clone of B lymphocytes. To further optimize diagnostic sensitivity, these studies should be complemented by measuring serum and urine free light-chains (sFLC and uFLC).

Despite the limitation of urinary testing, such as incorrect specimen handling, degeneration of urine proteins, and variability in FLC due to renal function [43], uFLC analysis can be a valuable tool in daily practice. The detection of uFLC in UIFE provides direct evidence of pathological light-chain production, with a complementary role to sFLC, due to its capacity to provide confirmatory diagnostic evidence, especially in ambiguous cases or when sFLC results are inconclusive [44,45]. Furthermore, UIFE was shown to be superior to the sFLC assay in detecting light-chains in amyloidosis [46]. Moreover, uFLC has been shown to have utility for monitoring therapeutic responses. A reduction of urinary monoclonal protein levels to 50% has been shown to indicate an improvement in the treatment of MM [47]. Therefore, this evidence highlights the valuable role of uFLC in the diagnosis and therapeutic monitoring of MGRS in clinical settings.

Furthermore, measuring FLC is highly useful, as it suggests clonality when the serum kappa to lambda ratio is abnormal. However, due to the substantial role of the kidney in clearing FLC, patients with abnormal glomerular filtration rates (GFR) may experience a modification of the normal serum FLC ratio. Therefore, the recommended range of the ratio is 0.37–3.17, compared to what is suggested in patients with normal renal function (0.26–1.65) [8,11,20,48,49,50,51,52,53].

Following the detection of MG, the search for an underlying B lymphocyte clone should include a bone marrow aspirate and/or biopsy, often supplemented with flow cytometry analysis and immunochemistry. In patients with monoclonal IgM or a high suspicion of lymphoma, investigating pathological lymph nodes may be necessary, as these patients may have a non-plasma cell B-cell lineage that is detectable only through a lymph node biopsy (Figure 8) [11,20].

## 5. Principles of MGRS Treatment

Traditionally, since MGRS is driven by the presence of B lymphocytes, the treatment of MGRS-related kidney disease typically focused on eradicating the malignant clone-producing monoclonal immunoglobulins (Ig), rather than directly treating the kidney lesion itself. Although these clones generally have a low risk of malignancy, treatment is required to preserve renal function or, in patients with advanced chronic kidney disease (ESRD) who are candidates for kidney transplantation, to prevent recurrence in the transplanted kidney [10,20]. However, individual evaluation is necessary, especially for elderly patients who may be frail and have comorbidities, to assess the expected benefit.

In recent times, it has become evident that nephrotoxic monoclonal immunoglobulin can damage the kidneys, regardless of the neoplastic burden. This has led to a substantial change in the goals of treatment, with a shift from exclusively tumor-based approaches to one that also takes into consideration the presence or absence of life-threatening conditions and the involvement of extrarenal organs. Therefore, preserving renal function has become a key factor to consider when determining the treatment for MGRS. Particularly, the use of conservative kidney treatments, including antihypertensive and antiproteinuric drugs, the correction of metabolic abnormalities, and supportive care, as well as dialysis or kidney transplantation for ESRD patients, is recommended. Moreover, the treatment should aim to balance potential benefits and risks for the patients, and to minimize renal toxicity. However, ESRD patients eligible for kidney transplantation are at increased risk of MGRS recurrence, which increases the importance of indicators of graft loss, such as the degree of hematologic remission and the recurrence rate and prognosis of the underlying disease. Therefore, hematologic treatment is one of the most crucial factors in preventing renal failure after kidney transplantation in patients with MGRS [54,55].

Rituximab is an appropriate treatment in cases induced by underlying lymphocytic or lymphoplasmacytic proliferation [15]. In plasma cell proliferative disorders, Bortezomib, a proteasome inhibitor, is currently the cornerstone of therapy [15]. Daratumumab, an anti-CD38 monoclonal antibody, has demonstrated high efficacy in patients with AL amyloidosis, and is currently being evaluated for the treatment of MIDD and PGNMID [56,57]. If its benefits, such as high response rates and relatively low rates of adverse effects, can be confirmed, this agent could be an excellent addition to the existing treatment regimens for plasma cell clones in patients with MGRS-related diseases.

One of the most severe and frequent complications associated with monoclonal Ig in the kidneys is the development of AL amyloidosis. Since patients with AL amyloidosis often also have cardiac involvement, and since heart disease is the leading cause of death in these patients, it is essential to evaluate for cardiac amyloidosis [15].

The first-line induction therapy for individuals diagnosed with AL amyloidosis is the combination of daratumumab, cyclophosphamide, bortezomib, and dexamethasone (Dara-CyBorD), established as the standard of care based on the ANDROMEDA trial [58,59]. An autologous stem cell transplant is performed on eligible patients, especially those who do not attain a satisfactory response to Dara-CyBorD, offering a survival advantage over other treatments [60,61,62,63]. Renal outcomes in patients with renal amyloidosis treated with chemotherapy were assessed by Pinney and Gillmore in a cohort of 429 patients. Their study found that 33% of patients showed a decrease in proteinuria and an increase in creatinine less than 25% from baseline, correlating with a significant reduction (>90%) in free light-chains (FLC) [64]. Furthermore, in patients who had also undergone renal transplantation, the 5-year survival rate of the transplanted kidney was 70%, with no recurrence of renal amyloidosis.

MIDD, predominantly caused by kappa light-chain deposition, is associated with a high risk of progressing to end-stage renal disease (ESRD) [21]. A study conducted on 56 patients with MIDD showed that 34% of patients treated with conventional chemotherapy progressed to chronic kidney disease, and the use of ASCT also resulted in nephropathy progression in 38% of cases. More recently, the introduction of Bortezomib-based therapy, associated with an overall hematologic response rate (Complete Response or CR, Very Good Partial Response or VGPR) exceeding 90%, has also shown excellent results in renal function. In particular, patients who achieved VGPR or higher had a greater likelihood of achieving a renal response compared to those who did not achieve any remission [65]. Bortezomib therapy has been highly effective, allowing for complete renal remission in the majority of patients, where other pharmacological agents, including Thalidomide, Lenalidomide, and other alkylating agents, were not as effective [66].

In summary, the treatment of MIDD should be based on the stage of chronic kidney disease at onset [15]. For chronic kidney disease stages 1–3, the primary goal is to preserve renal function, and initial therapy should include Bortezomib, followed by high-dose Melphalan with ASCT in appropriately selected patients without extrarenal manifestations. For patients who are not eligible for ASCT, therapy with Bortezomib alone may be proposed, as it has been shown to improve renal responses, especially in patients with a pre-therapy GFR > 30 mL/min/1.73 m^2^ and a difference in free light-chain levels pre- versus post-treatment of <40 mg/L. In chronic kidney disease stages 4 and 5, the likelihood of recovering renal function is low; therefore, treatment is not recommended unless there is extrarenal involvement, or the patient needs to be referred for a kidney transplant. However, it should be emphasized that the recurrence rate of nephropathy after kidney transplantation is high, so it is essential to achieve a complete hematological response before initiating kidney transplantation [21,28,65].

Tubular lesions resulting from the deposition of light-chains can manifest as cytoplasmic crystalline or non-crystalline light-chain deposits [22,42]. Apart from metabolic complications (osteomalacia, tubular acidosis), this syndrome has a slow progression, and most patients do not reach ESRD because they typically succumb to other causes [67]. Treatment has no significant effects on these patients. Due to the indolent nature of this disorder, some experts suggest that treatment should be primarily supportive, including electrolyte supplementation to prevent osteomalacia, due to the uncertain risk–benefit ratio [67]. In fact, in clinical trials conducted a few years ago, patients treated with alkylating agents experienced significant side effects, with about one-fifth of deaths resulting from secondary acute leukemias or myelodysplastic syndromes [68]. Therefore, even in these cases, the use of newer generation drugs, such as Bortezomib, Thalidomide, and Lenalidomide, in combination with stem cell transplantation, has been proposed. The safety of these new chemotherapy agents and stem cell transplantation has made it possible to preserve renal function in patients with mild chronic kidney disease, especially when diagnosed early [69,70,71,72,73,74,75]. However, in patients with advanced-stage chronic kidney disease who are not eligible for kidney transplantation and do not have multiple myeloma, the risks of such treatments may outweigh the benefits [15].

Treatment for less common nephropathies associated with MGRS, for instance, membranoproliferative glomerulonephritis, immunotactoid glomerulonephritis, and PGNMID, aims to eradicate the malignant clone [15]. The choice of chemotherapy strategy depends on whether the clone is considered to be of lymphocytic or plasma cell origin.

## 6. Conclusions

Kidney diseases related to the presence of paraproteinemia encompass a wide range of renal lesions resulting from the presence of MGRS, multiple myeloma, and Waldenström macroglobulinemia. Although their clinical presentation and histology differ, their progressive nature and tendency to recur after kidney transplantation are common. Renal biopsy is necessary for diagnosis, and prior to cytostatic treatment, determining the pathogenicity of the monoclonal protein is essential. Treatment should aim to eradicate the pathological clone responsible for producing the nephrotoxic monoclonal component.

## Figures and Tables

**Table 1:** Features of clonal proliferative disorders involving B lymphocytes and plasma cells.

Disease	Clone	Bone Marrow	Immunoglobulins	M Spike	Organ Damage
MGUS	Any clone	<10%	Any type	<30 g/L	None
Smoldering MM	Plasma cell	10–60%	Any type	≥30 g/L	None
MM	Plasma cell	≥10%	Any type	≥30 g/L	Hypercalcemia, Renal Failure, Anemia, and Bone Lesions (CRAB)
Smoldering WM	Lymphoplasmocytic lymphoma clone	≥10%	IgM	≥30 g/L	Absent
WM	Lymphoplasmocytic lymphoma clone	≥10%	IgM	≥30 g/L	Anemia, Blood Hyperviscosity, Hepatosplenomegaly, Lymphadenopathy, Neuropathy, and Systemic Symptoms
MBL	B-cell clone	Peripheral B-cell count < 5 × 10^9^/L	Any type	Any level	Lack of Lymphnode involvement
CLL	B-cell clone	Peripheral B-cell count > 5 × 10^9^/L	Any type	Any level	Lymphadenopathy, Anemia, and Thrombocytopenia
Other B-cell lymphoproliferative disorders	Pan B-cell markers (CD19^+^ CD20^+^ CD79^+^ CD22^+^ PAX5^+^)	Presence or absence	Any type	Any level	Lymphadenopathy, Splenomegaly

CLL, chronic lymphocytic leukemia; MBL, monoclonal B-cell lymphocytosis; MGUS, monoclonal gammopathy of undetermined significance; MM, multiple myeloma; WM, Waldenström macroglobulinemia.

**Table 2:** Pathogenetic mechanisms of renal damage in monoclonal gammopathies.

Possible Pathogenetic Mechanisms Involved in Renal Damage During Monoclonal Gammopathies
Intraparenchymal deposition of circulating light-chains Precipitation of intratubular casts during the excretion phase of light-chains Intracytoplasmic accumulation of light-chains in proximal tubule epithelium with “Fanconi-like” syndrome Release of membrane-permeabilizing factors affecting the glomerular basement membrane Injuries secondary to tumor lysis (e.g., Uric Acid Nephropathy) Renal stones associated with hypercalcemia Iatrogenic damage (e.g., due to the use/abuse of NSAIDs ^1^) Direct infiltration of renal tissue by monoclonal plasma cells

^1^ NSAIDs, non-steroidal anti-inflammatory drugs.

**Table 3:** Histopathological characteristics of renal lesions detected in renal biopsy in MGRS.

Organized Monoclonal Ig Deposits		Pathological Findings
Fibrils	Ig-related Amyloidosis (see Figure 4)	
	Light Microscopy	Mesangial expansion due to the accumulation of amorphous/acellular material, weakly PAS-positive, poorly argyrophilic, nodular in more advanced forms. Often coexisting deposits along the GBM in subendothelial or subepithelial locations (in the form of coarse “flame” spikes). Frequent involvement of arteriolar vessels and interstitium. Congo-red-positive.
	Immunofluorescence	Intense expression of a single light-chain in AL amyloidosis (in more than 75% of cases, lambda chains are found); intense expression of a single heavy Ig chain (most commonly gamma) with negative light-chains in AH amyloidosis; intense expression of a single heavy Ig chain and a single light-chain in AHL amyloidosis.
	Electron Microscopy	Non-branching, randomly oriented fibrils; 8–12 nm in diameter.
	Monoclonal Fibrillary GN (FGN)	
	Light Microscopy	Variable aspects of mesangial, membranous, or membranoproliferative GN; Congo-red-negative.
	Immunofluorescence	More frequently, polyclonal deposits of Ig, typically IgG.
	Electron Microscopy	Non-branching, randomly oriented fibrils; 12–24 nm in diameter.
Microtubules	Immunotactoid GN (ITGN)	
	Light Microscopy	Variable aspects of mesangial, membranous, or membranoproliferative GN; Congo-red-negative.
	Immunofluorescence	Glomerular deposits of light-chains; possible positivity for Ig (most commonly IgG1) and C3.
	Electron Microscopy	Electron-dense deposits in mesangial, subepithelial, or subendothelial areas with a structured appearance in the form of hollow microtubules arranged in parallel, usually with a diameter greater than 30 nm.
	Type 1 and 2 Cryoglobulinemic GN	
	Light Microscopy	Predominantly membranoproliferative or endocapillary proliferative GN aspect; often large intraluminal eosinophilic deposits (“pseudo-thrombi”); intensely PAS-positive with a vitreous appearance.
	Immunofluorescence	Monoclonal light- and heavy-chains (most commonly IgGk) and complement.
	Electron Microscopy	Capillary lumina segmentally occluded by large subendothelial and intracapillary electron-dense deposits; may appear organized in more than half of the patients.
Crystalline Inclusions Or Deposits	Light-Chain Proximal Tubulopathy (LCPT) (see Figure 5)	
	Light Microscopy	Atrophy and dedifferentiation of proximal tubular cells (PTC), swelling of PTC cytoplasm.
	Immunofluorescence	Presence of lambda or kappa light-chains in PTC.
	Electron Microscopy	Amorphous granular accumulation of light-chains; increased lysosomal volume with a spotted appearance.
	Crystal-Storing Histiocytosis (CSH)	
	Light Microscopy	Histiocytes with crystalline inclusions in the interstitium and perirenal adipose tissue; atrophy and dedifferentiation of PTC.
	Immunofluorescence	Light-chain inclusions in PTC, especially kappa.
	Electron Microscopy	Needle-like crystals within histiocytes and occasionally in PTC and glomerular cells.
	(Cryo) Crystalglobulin-associated GN (CGN)	
	Light Microscopy	Large intra-arteriolar and intraglomerular thrombi consisting of Ig, peri-vascular inflammatory infiltrate.
	Immunofluorescence	Intravascular Ig inclusions.
	Electron Microscopy	Crystalline structure or periodicity of intravascular thrombi.
Not organized monoclonal Ig deposits		Pathological findings
Monoclonal immunoglobulin deposition disease (MIDD) (see Figure 6)		
Light Microscopy		Global mesangial nodular sclerosis, PAS-positive; thickening of the tubular basement membrane (TBM) may coexist.
Immunofluorescence		Diffuse linear deposition of monoclonal proteins along the glomerular basement membrane (GBM) and the tubular basement membrane (TBM) (light-chains only for LCDD, light- and heavy-chains for LHCDD, a single class of Ig for HCDD).
Electron Microscopy		Presence of punctate electron-dense deposits in a “salt and pepper” pattern on the inner side of GBM and the outer side of TBM.
Proliferative Glomerulonephritis with Monoclonal IgG Deposits (PGNMID) (see Figure 7)		
Light Microscopy		Membranoproliferative GN, with diffuse and global double contours at glomerular capillary wall with mesangial expansion.
Immunofluorescence		Coarse granular glomerular deposits of monoclonal immunoglobulins or, more rarely, monoclonal light-chains.
Electron Microscopy		More frequently, subendothelial and mesangial electron-dense deposits; less frequently, sub-epithelial deposits.
Not monoclonal Ig deposits		Pathological findings
C3 Glomerulonephritis associated with Monoclonal Gammopathy (MG-C3GN)		
Light Microscopy		Membranoproliferative GN; mesangial proliferation; signs of endocapillary proliferation may coexist.
Immunofluorescence		Prevalent deposits of C3 in the mesangium and in the capillary wall; little or no Ig deposits.
Electron Microscopy		Intramembranous deposits and large, rounded electron-dense mesangial deposits in DDD (dense deposit disease); poorly defined mesangial, intramembranous, and subendothelial deposits in C3GN.
Thrombotic Microangiopathy (TMA)		
Light Microscopy		Endocapillary thrombi associated with mesangial expansion and endothelial proliferation, sometimes mesangiolysis, thinning of the subendothelial zone, and double contours of the glomerular basement membrane.
Immunofluorescence		Lack of monoclonal immune deposits.
Electron Microscopy		Detachment of the endothelium from the basement membrane and presence of electron-transparent material and cellular debris in the subendothelial space; demining of the GBM.

AH, amyloidosis, heavy-chain amyloidosis; AHL, amyloidosis, heavy-/light-chain amyloidosis; AL, amyloidosis, light-chain amyloidosis; CGN, cryoglobulinemic GN; CSH, crystal-storing histiocytosis; DDD, dense deposit disease; FGN, fibrillary GN; GBM, glomerular basement membrane; GN, glomerulonephritis; HCDD, heavy-chain deposition disease; Ig, immunoglobulin; ITGN, immunotactoid GN; LCDD, light-chain deposition disease; LCPT, light-chain proximal tubulopathy; LHCDD, light- and heavy-chain deposition disease; MG-C3GN, C3 GN associated with monoclonal gammopathy; MIDD, Monoclonal Ig Deposition Disease; PAS, periodic acid-Schiff; PGNMID, proliferative GN with monoclonal Ig deposits; PTC, proximal tubular cell; TBM, tubular basement membrane; TMA, Thrombotic Microangiopathy.

## Data Availability

No new data were created or analyzed in this study. Data sharing is not applicable to this article.
